# Drought-Stress Tolerance in Wheat Seedlings Conferred by Phenazine-Producing Rhizobacteria

**DOI:** 10.3389/fmicb.2019.01590

**Published:** 2019-07-10

**Authors:** Tessa Rose Mahmoudi, Jun Myoung Yu, Shuyu Liu, Leland S. Pierson, Elizabeth A. Pierson

**Affiliations:** ^1^Department of Plant Pathology and Microbiology, Texas A&M University, College Station, TX, United States; ^2^Department of Applied Biology, Chungnam National University, Daejeon, South Korea; ^3^Texas A&M AgriLife Research and Extension Center, Amarillo, TX, United States; ^4^Department of Horticultural Sciences, Texas A&M University, College Station, TX, United States

**Keywords:** drought-stress tolerance, phenazine, *Pseudomonas*, wheat, plant–microbe interactions

## Abstract

The specific role of phenazines produced by rhizosphere-colonizing *Pseudomonas* in mediating wheat seedling drought-stress tolerance and recovery from water deficit was investigated using *Pseudomonas chlororaphis* 30-84 and isogenic derivatives deficient or enhanced in phenazine production compared to wild type. Following a 7-day water deficit, seedlings that received no-inoculum or were colonized by the phenazine mutant wilted to collapse, whereas seedlings colonized by phenazine producers displayed less severe symptoms. After a 7-day recovery period, survival of seedlings colonized by phenazine-producing strains exceeded 80%, but was less than 60% for no-inoculum controls. A second 7-day water deficit reduced overall survival rates to less than 10% for no-inoculum control seedlings, whereas survival was ∼50% for seedlings colonized by phenazine-producers. The relative water content of seedlings colonized by phenazine-producers was 10–20% greater than for the no-inoculum controls at every stage of water deficit and recovery, resulting in higher recovery indices than observed for the no-inoculum controls. For 10-day water deficits causing the collapse of all seedlings, survival rates remained high for plants colonized by phenazine-producers, especially the enhanced phenazine producer (∼74%), relative to the no-inoculum control (∼25%). These observations indicate that seedlings colonized by the phenazine-producing strains suffered less from dehydration during water deficit and recovered better, potentially contributing to better resilience from a second drought/recovery cycle. Seedlings colonized by phenazine-producing strains invested more in root systems and produced 1.5 to 2 fold more root tips than seedlings colonized by the phenazine mutant or the no-inoculum controls when grown with or without water deficit. The results suggest that the presence of phenazine-producing bacteria in the rhizosphere provides wheat seedlings with a longer adjustment period resulting in greater drought-stress avoidance and resilience.

## Introduction

Maintaining crop productivity under drought-stress conditions is an important concern that threatens global food production (Daryanto et al., 2016; Fahad et al., 2017). Elevated temperatures predicted from climate change will increase the rate of soil drying in agricultural land, resulting in more rapid onset of drought stress with higher intensity (Trenberth et al., 2014; Fischer and Knutti, 2015). Thus, it is important to improve the ability of plants to withstand prolonged water deficits and rapidly adapt to and recover from them.

Plant drought-stress tolerance, the ability of plants to maintain productivity during periods of drought-stress, is a complex phenotype (Ngumbi and Kloepper, 2016). Plants have numerous mechanisms to tolerate drought-stress at the organ system to cellular levels, including adaptations in plant growth and development resulting in alterations in plant allometry, root system development, and even time to flowering; osmotic adjustment and optimization of water use; and induction of stress-responsive pathways, including management of reactive oxygen species (ROS) (Farooq et al., 2009; Osakabe et al., 2014; Meena et al., 2017). With regard to plant growth and development, it is now well recognized that alterations in root system architecture (RSA), especially the proliferation of higher order roots (and thus root tips essential for the uptake of water and nutrients), aids in short-term adaptation to water deficit (Robinson et al., 1991; Comas et al., 2013; Xu et al., 2015). To date, improvements in plant drought-stress tolerance have been addressed primarily by breeding directly for desired plant phenotypes. However, plants do not experience drought-stress in isolation. Plant-associated microbial communities may enhance plant drought-stress tolerance leading to improved crop productivity under drought conditions.

Plant growth-promoting rhizobacteria (PGPR), plant-beneficial bacteria inhabiting the plant rhizosphere, have the potential to enhance plant adaptation to drought directly or indirectly. Examples of PGPR direct effects include promoting changes in plant growth and development (especially RSA) to enhance water and nutrient uptake, altering stomatal regulation, producing compatible solutes, mediating redox stress, producing or catabolizing metabolites that act as plant hormones, producing volatile organic compounds, or tapping into other plant stress response pathways (Berendsen et al., 2012; Farag et al., 2013; Nadeem et al., 2014; Ngumbi and Kloepper, 2016; Naylor and Coleman-Derr, 2018). Additionally, PGPR may provide important indirect benefits to plant growth and development via improved soil structure and increased soil water retention (Chang et al., 2007; Naseem and Bano, 2014; Le Tourneau et al., 2018). Moreover, evidence suggests that plants recruit specific microbial communities to their rhizospheres (Bergsma-Vlami et al., 2005; el Zahar Haichar et al., 2008; Mendes et al., 2014; Yan et al., 2017). This occurs primarily through alterations in rhizodeposition patterns, including carbon and nitrogen inputs (Rovira, 1959; Latour et al., 1996). Rhizodeposition is a temporally and spatially variable trait influenced by plant genotype (Rengel et al., 1998; Miethling et al., 2000; Berg et al., 2002, 2006; Kuklinsky-Sobral et al., 2004; Mazzola et al., 2004; İnceoğlu et al., 2012) and environmental conditions (Baudoin et al., 2003; Henry et al., 2007; Song et al., 2012; Calvo et al., 2017). In other words, plants may recruit higher populations of rhizosphere microorganisms under certain conditions, and evidence suggests that this selection is highly dependent on the functional capacities rather than the taxonomy of these communities (Mendes et al., 2014; Yan et al., 2017). Understanding the influence of microbial functionalities that are both prevalent and beneficial in dryland agriculture will provide opportunities to take advantage of these plant–microbe interactions to improve plant productivity in dryland agriculture.

Phenazines are a class of diffusible, heterocyclic compounds that differ from one another based on the substitution of various functional groups on the core phenazine ring structure (Mavrodi et al., 2001, 2006; Biessy and Filion, 2018). Among PGPR strains, phenazine production has been shown to contribute to inhibition of a diversity of plant pathogens and suppression of the plant diseases they cause (Thomashow and Weller, 1988; Chin-A-Woeng et al., 2001; Mavrodi et al., 2006; Pierson et al., 2010; Cezairliyan et al., 2013; Zhou et al., 2016) and the rhizosphere competence of producers (Mazzola et al., 1992; Chin-A-Woeng et al., 2003). Phenazines also contribute to biofilm formation and architecture and enhance extracellular matrix production (Maddula et al., 2006, 2008; Das T. et al., 2015; Wang et al., 2016). Phenazines are redox active metabolites that have been studied extensively in human pathogens due largely to their capacity to generate host-damaging ROS (Mavrodi et al., 2001; Price-Whelan et al., 2006; Pierson et al., 2010). The specific role of bacterial phenazine production in plant drought-stress tolerance has not been tested.

Phenazine-producing bacteria have been reported to be prevalent in the rhizospheres of wheat grown under dryland production conditions. For example, Mavrodi D.V. et al. (2012) reported that in Washington, United States, indigenous pseudomonads having phenazine biosynthetic genes were detected at higher frequencies and greater numbers on roots of winter wheat grown in dryland production as compared to irrigated production. These studies focused on the occurrence of *Pseudomonas* strains with the genes for the production of bacterial secondary metabolites linked to plant disease suppression, specifically phenazines or 2,4-diacetylphloroglucinol. It also was reported that the frequency of wheat root systems colonized by pseudomonads having phenazine biosynthetic genes was inversely related to annual precipitation, suggesting that phenazine-producing pseudomonads do better in the rhizospheres of wheat experiencing low soil moisture (Mavrodi O.V. et al., 2012). More recently, Mavrodi et al. (2018) demonstrated that 3 years of overhead irrigation applied to a dryland production site in Washington significantly reduced the frequency and size of wheat rhizosphere populations of indigenous pseudomonads having phenazine biosynthetic genes relative to adjacent non-irrigated plots (Mavrodi et al., 2018). In Texas, Mahmoudi (2017) reported that populations of pseudomonads having phenazine biosynthetic genes were generally higher in the rhizospheres of the drought-tolerant cultivars TAM 111 and TAM 112 compared to the drought-sensitive cultivar TAM 304. This trend was most pronounced when the cultivars were grown in soil collected from a long-term dryland production site, as compared to soil collected from an adjacent long-term irrigated production site. These results reinforce the hypothesis that wheat may select this bacterial functionality, phenazine production, under low soil moisture conditions.

In the present study, we examined the role of phenazines produced by *Pseudomonas* in mediating wheat seedling drought-stress tolerance and recovery from cycles of prolonged water deficit. Investigation of the specific role of bacterial phenazine production was facilitated by having isogenic derivative strains of *Pseudomonas chlororaphis* 30-84 deficient or enhanced in phenazine production compared to the wild type (Yu et al., 2018b). The focus was on bacterially mediated improvements in wheat seedling drought-stress tolerance because seedling establishment is often the most vulnerable stage and may have large impacts on crop stand and yield (Liao et al., 2006). For these experiments, TAM 112 wheat seedlings were grown in soil inoculated either with enhanced or wild type phenazine producers or the phenazine mutant, or with no inoculum. We were particularly interested in determining whether any potential benefits to wheat seedlings associated with bacterial phenazine production would be observed under both well-watered and water-deficit conditions.

## Materials and Methods

### Plant and Soil Material

Soil used for these experiments was a Pullman clay loam^1^ and collected from the USDA-ARS, Bushland, TX dryland wheat plots at a depth of 1 to 15 cm. Prior to use in pots, it was necessary to sieve (2 mm mesh) and mix the soil with sand (soil: sand, 2:1, v:v) to facilitate drainage. The soil-sand mix, hereafter referred to as soil, was autoclaved twice (121°C, 15 PSI, 1 h, 24 h pause between cycles). The hard red winter wheat cultivar TAM 112 developed by Texas A&M AgriLife Research for use in dryland production (Reddy et al., 2014; Rudd et al., 2014) was used for all studies.

### Bacterial Inoculum

The bacterial strains and plasmids used in this study are described in Table 1. A spontaneous rifampicin-resistant derivative of *P. chlororaphis* 30-84 was used in all studies and is hereafter referred to as wild type (30-84WT). Also used was a derivative of *P. chlororaphis* 30-84 (30-84Enh) with enhanced phenazine production obtained via a 90-bp deletion of sequence within the 5′-untranslated region (5′-UTR) of *phzX*, the first gene in the phenazine biosynthetic operon (Yu et al., 2018b). A phenazine deficient mutant (30-84ZN) (Wood et al., 1997), was employed as a control. *P. chlororaphis* 30-84 produces three phenazines: phenazine-1-carboxylic acid (PCA), 2-hydroxy-phenazine-1-carboxylic acid (2OHPCA) and a small amount of 2-hydroxy-phenazine (2OHPHZ). The two, 2-hydroxy phenazines are produced from PCA (the product of the phenazine operon *phzXYFABCD*) via the activity of a phenazine-modifying enzyme (monooxygenase) encoded by *phzO* (Delaney et al., 2001; Pierson et al., 2010). In one experiment, derivatives of 30-84 producing pyocyanin (30-84MS) or phenazine-1-carboxamide (30-84H) were used (Table 1). These derivatives were obtained via overexpression of *P. aeruginosa* genes encoding the enzymes responsible for the conversion of PCA to pyocyanin (*phzM* and *phzS)* or phenazine-1-carboxamide (*phzH) in trans* in 30-84PCA, a Δ*phzO P. chlororaphis* 30-84 mutant (Yu et al., 2018a). *P. chlororaphis* 30-84 and its derivatives were grown at 28°C in Luria-Bertani medium (LB) medium containing 5 g of NaCl per liter (Lennox, 1955) or King’s Media B (KMB) (King et al., 1954). Antibiotics were used where appropriate at the following concentrations: gentamicin (Gm) at 50 μg/ml, rifampicin (Rif) at 100 μg/ml, kanamycin (Km) at 50 μg/ml, or cycloheximide (CM) 100 μg/ml.

**TABLE 1:** Bacterial strains and plasmids used in this study.

**Strain**	**Description^a^**	**Reference or source**
***P. chlororaphis***		
30-84WT	PCA^+^, 2-OH-PCA^+^, 2-OH-PHZ^+^, Rif^R^, Wild type	Pierson et al.,1992
30-84Enh	PCA^+^, 2-OH-PCA^+^, 2-OH-PHZ^+^, Rif^R^, 90 bp deletion at the 5’UTR of *phzX*	Yu et al.,2018b
30-84ZN	Phz^–^, *phzB*::*lacZ*, Rif^R^	Wood et al.,1997
30-84PCA	PCA^+^, *phzO*::Tn5, Km^R^, Rif^R^	Maddula et al.,2008
30-84H	PCA^+^, PCN^+^, 30-84PCA containing plasmid pGT2-*phzH*, Rif^R^	Yu et al.,2018a
30-84MS	PCA^+^, PYO^+^, 30-84PCA containing plasmid pGT2-*phzMS*, Rif^R^	Yu et al.,2018a
**Plasmid**		
pGT2	pProbe-GT’: pVS1 replicon, p15a origin of replication, *gfp* transcriptional fusion; Gm^R^	Miller et al.,2000

*^*a*^Km^*R*^, Gm^*R*^, and Rif^*R*^ = kanamycin, gentamicin, and rifampin resistance, respectively.*

Inoculum of each of the strains was prepared by growing them separately in LB broth (24 h, 28°C, rapid agitation). Cells were pelleted and washed three times with sterilized deionized water, and bacterial populations were adjusted to the same final optical density (OD_620_ of 0.8) in sterile water. The autoclaved soil was inoculated with bacteria by mixing the inoculum thoroughly with the soil (1.5 ml inoculum in 20 ml water to 500 g soil) and allowing bacterial populations to equilibrate in soil for 4–6 days before being parsed into separate containers for planting; mean population densities for the three derivatives were checked following equilibration to insure they were sufficient (ca. 10^7^–10^8^ CFU/g of soil) for every experiment. For the no-inoculum control, the same volume of sterilized water was used to treat the soil.

### Plant Growth and Development Assay – Non-stressed Conditions

Wheat seedlings were grown in 1 gallon pots (13 cm diameter, 15 cm height) containing soil (2100 g) inoculated either with 30-84Enh, 30-84WT, 30-84ZN, or sterile water (no-inoculum control). Prior to planting, wheat seeds were surface sterilized using 0.6% NaClO for 10 min, followed by multiple rinses in sterile-distilled water. Seeds were pre-germinated on sterilized germination paper and covered with autoclaved vermiculite. A total of 20 plants/pot and 4 pots/treatment were arranged in a randomized complete block design (4 blocks) and watered every 3 days for 3 weeks with 50 ml sterile deionized water. Seedlings were grown 21 days in a growth chamber (16^∘^C, 12:12 light/dark cycle, ∼50% RH).

Seedlings then were harvested and roots were washed to remove adhering soil. Bacterial population sizes were determined from a random sample of replicates via serial dilution plating on LB agar supplemented with rifampicin. Plant growth and development was assessed by measuring root, shoot, and whole plant dry weight production. Root system metrics were obtained using WinRhizo (*Arabidopsis* Version, Regent Instruments, Inc., Quebec, Canada) to analyze scanned (EPSON Perfection V700) images of the root systems. For root scans, freshly harvested roots were floated in ∼1 cm water in the clear plastic containers supplied for use with the WinRhizo system. Roots were carefully arranged to minimize overlap before scanning and Photoshop was used to remove soil particles, shadows, or other imperfections in the images. WinRhizo software provided estimates of total root length and surface area and number of root tips. Dry weights were obtained after drying plants (65^∘^C, 72 h).

### Drought-Stress Tolerance Assay

Wheat seedlings were grown in plastic tubes (Ray Leach *Cone-tainers*, 2.5-cm diameter × 16.5-cm long) containing soil inoculated either with 30-84Enh, 30-84WT, 30-84ZN, or sterile water (no-inoculum control). Prior to planting, wheat seeds were surface sterilized and germinated on sterilized germination paper as described above, and two 2-day-old seedlings were sown into each container and covered with autoclaved vermiculite. After 2 days establishment, seedlings were thinned to 1 plant/container. A total of 60 plants per treatment were arranged in a randomized complete block design (4 blocks × 15 reps).

Wheat seedlings were grown unstressed for 21 days (16^∘^C, 12:12 light/dark cycle, ~50% RH) and watered every 3 days with 5 ml sterile deionized water. Bacterial populations on randomly selected replicates were determined via serial dilution as described above. After this establishment period, seedlings received either a “moderate” water deficit treatment (water withheld for 7 days, all no-inoculum control plants severely wilted with significant leaf browning) or, in a separate experiment, a “severe” water-deficit treatment (water withheld for 10 days, all seedlings exhibited severe wilting and browning – 10 days was determined from preliminary experiments to be the longest water deficit interval from which seedlings were able to recover). All plants were then watered (5 ml, every 3 days) and after 7 days recovery, recovery index (RI) and survival rate were determined. RI was based on the amount of above ground tissue recuperated after wilting (RI-0 = no recovery, RI-1 = slight new growth/recovery of leaves, RI-2 = partial recovery of leaves, RI-3 = recovery of one or more entire leaves). Plants receiving an RI rating of 0 were considered dead. Following the 7-day recovery period, the seedlings in both the moderate and severe water-deficit experiments were exposed to a second cycle of water deficit (7 days) and recovery (7 days) and recovery index and survival rate determined. At the end of the second cycle of water deficit and recovery, plants were carefully harvested to preserve the root system and plant growth and development measurements, including root system metrics were obtained as described above.

Additionally, for the moderate water-deficit experiment (water withheld for 7 days), subsets of plants from each treatment were sampled on days 1, 3, and 7 of the first drought cycle and on the last/seventh day of first recovery cycle (day 14) to determine the relative health of the drought-stressed seedlings as inferred from the relative water content of the seedlings. Plants were harvested carefully to preserve the root systems. Individual intact plants were weighed separately to measure fresh weight, wrapped in paper towel and allowed to soak for 16 h in sterile deionized water. Plants then were blotted dry and weighed again in order to obtain turgor weights. Plants then were oven dried (65°C, 96 h) to obtain dry weights. The relative water content of seedlings was calculated using the equation: RWC(%) = [(FW–DW)/(TW–DW)] × 100, where FW = fresh weight determined at the time of harvest, DW = dry weight determined after oven drying, and TW = turgor weight obtained after immersing plant tissue in sterile water (Turner, 1981).

The severe water deficit experiment (water withheld for 10 days) was repeated on vernalized plants grown to the jointing stage, i.e., the stage when the first joint (node) emerges above the soil line, which coincides with the initiation of the reproductive phase of development. Prior to vernalization, surface sterilized winter wheat seeds were sown in soil inoculated either with 30-84Enh, 30-84WT, or 30-84ZN, or no inoculum (control). After germination (4 days post- planting), seedlings were vernalized (5°C, 8 weeks). Following vernalization, bacterial populations on several replicates were determined via serial dilution and the remaining seedlings were transferred to large plastic tubes (Ray Leach *Cone-tainers*, 4 cm diameter, 21 cm height) containing soil with the same soil inoculation treatment as used pre-vernalization. Plants were watered with sterile deionized water (10 ml, every 3 days). During this time plants were maintained under the same growth conditions described above. Plants then received no water for 15 days, and seedlings were harvested and measured as above.

### Growth Assay in Natural Soil

To determine whether phenazine-producing bacteria could promote root growth in natural soil, root production was measured using a repeated plant/harvest cycle assay as described previously (Yu et al., 2018a). The phenazine-producing strains used in this experiment included 30-84Enh, 30-84WT, 30-84MS, and 30-84H. The phenazine non-producing strain 30-84ZN was included as a control. Briefly, bacterial inoculum was prepared in KMB broth and inoculated into sieved Bushland field soil (soil:sand, 2:1, v:v) as described previously (Yu et al., 2018a); soil populations following the equilibration period were ∼10^8^ CFU/g of soil for all treatments. Wheat seeds were surface sterilized and germinated as described previously (Yu et al., 2018a). After 3 days, the germinated seedlings were planted into plastic tubes (Ray Leach *Cone-tainers*, 2.5-cm diameter, 16.5-cm long) containing the inoculated soil mix. The tubes (60 tubes per treatment) were arranged in a randomized complete block design. Plants were maintained on a light shelf in a plant-growth room (27 ± 2°C, 16:8 light/dark, ∼50% RH) and were watered with sterile distilled water (5 ml, every 3 days). Each plant/harvest cycle lasted 21 days, and 6 plants per treatment were harvested at the end of each plant/harvest cycle. Bacterial populations on roots were determined by serial dilution plating on LB amended with rifampicin and cycloheximide (fungicide), and root dry weight biomass was determined. For the plants not used in the CFU estimations, shoots were removed at the soil surface level. The soil and remaining root systems were homogenized and the processed soil was reused as the planting medium for the next plant/harvest cycle, as previously described (Mazzola et al., 1992; Yu et al., 2018a). Newly germinated wheat seedlings were re-planted into the processed soil and the plant/harvest/sample cycle was repeated 5 times.

### Statistical Analyses

All data presented are the mean ± the standard error of the mean (SEM). Data were analyzed by ANOVA and Fisher’s protected Least Significant Difference (LSD) or Tukey test (*P* < 0.05) with GraphPad Prism software (GraphPad Software, San Diego, CA, United States).

## Results

### Rhizosphere Colonization by Phenazine-Producing Bacteria Promoted Seedling Investment in Roots and Altered RSA Under Non-stressed Conditions

To determine whether root colonization by phenazine-producing strains altered seedling growth and development, wheat seedlings were grown 21 days in soil inoculated either with 30-84Enh, 30-84WT, or 30-84ZN, or no inoculum. Mean population densities for the three bacterial strains in soil following equilibration were approximately 10^8^ CFU/g of soil. Mean bacterial population densities established on roots at day 21 were 10^7^–10^8^ CFU/g of root dry weight and there were no significant differences among bacterial treatments.

**FIGURE 1:**
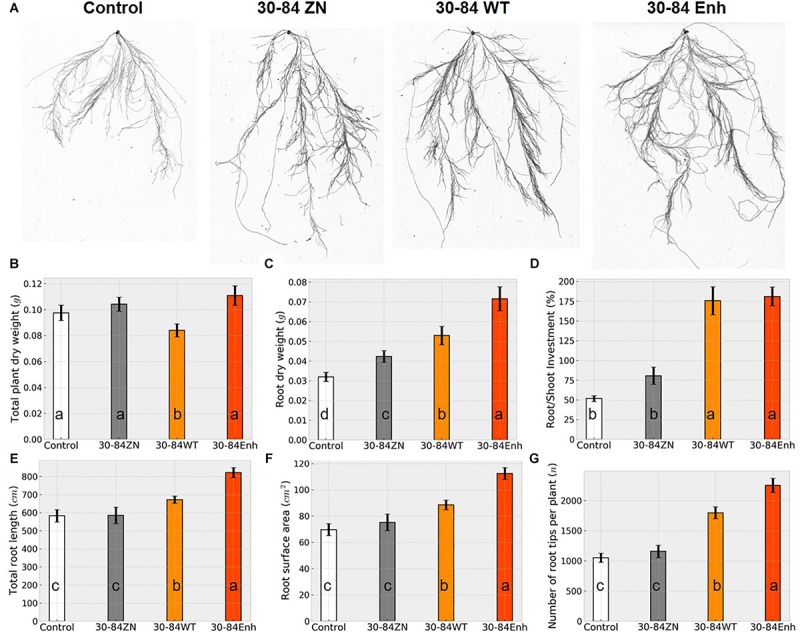
Colonization by phenazine-producing strains enhanced seedling investment in roots and altered root system architecture under non-stressed conditions. Wheat seedlings were grown in 1-gallon pots containing autoclaved soil inoculated either with 30-84Enh, 30-84WT, or 30-84ZN, or no inoculum (control). Seedlings were grown 21 days. **(A)** Representative images of root scans for each treatment. Plant growth characteristics including: **(B)** whole plant dry weight, **(C)** root dry weight and **(D)** root/shoot investment (based on dry weights), and root system characteristics obtained from WinRhizo software including, **(E)** total root length, **(F)** root surface area, and **(G)** number of root tips per plant. Data are the mean and standard error (*N* = 18 plants). Values with the same letter do not differ statistically as determined by a Fishers protected Tukey test (*P* > 0.05).

Although seedlings differed slightly in whole plant dry weight biomass, the root dry weight biomass and root to shoot investment (root/shoot dry weight ratio, as a percentage) of seedlings colonized by 30-84WT and 30-84Enh were greater than for non-treated seedlings, and these differences were more pronounced for the seedlings colonized by the phenazine-overproducer 30-84Enh (Figures 1A–D). For plants colonized by 30-84WT and 30-84Enh, seedling investment in the root system was over 1.5 times greater than investment in the shoot system (∼175%) as compared to ∼50% investment in the root versus shoot system for the no-inoculum seedlings (Figure 1D). For the phenazine non-producing control the average seedling investment in root compared to shoot system was ∼80%, demonstrating some alteration in biomass allocation compared to the no-inoculum control. These differences in investment in roots by seedlings colonized with phenazine-producing bacteria translated into significantly greater total root length, root surface area, and number of root tips, compared to the no-inoculum control, with root tip production being 1.5 to 2 fold greater for the seedlings colonized by the phenazine-producing strains (Figures 1E–G). The differences were most pronounced in seedlings colonized by the phenazine-overproducer 30-84Enh, whereas values for seedlings colonized by phenazine non-producer, 30-84ZN, were generally similar to those of the no-inoculum controls. Given the importance of root tips for water uptake, the greater investment in roots, especially in the production of higher order roots leading to greater proliferation of root tips, suggested that root colonization by phenazine-producing strains resulted in increased water uptake capacity by the seedling root systems.

### Rhizosphere Colonization by Phenazine-Producing Bacteria Improved Seedling Survival and Recovery Following Water Deficit Conditions

To determine whether bacterially mediated alterations in RSA enhanced seedling survival from drought stress, wheat seedlings were grown under well-watered conditions for 21 days in soil either inoculated with 30-84Enh, 30-84WT, or 30-84ZN, or no inoculum, and then water-deficit stress was induced by the cessation of watering. Seedlings received either a “moderate” water-deficit treatment (water withheld for 7 days, all no-inoculum control plants severely wilted) or, in a separate experiment, a “severe” water-deficit treatment (water withheld for 10 days, all seedlings exhibiting severe wilting). The effects of bacterial treatment on vernalized plants under severe water-deficit conditions also were examined in a separate experiment. As in the previous non-stressed experiment, in all experiments mean bacterial population densities at day 21 (prior to imposing the water deficit) were 10^7^–10^8^ CFU/g of root dry weight and there were no significant differences among the bacterial treatments. Similarly, there were no significant differences among the bacterial treatments in mean root population densities on vernalized plants on the initiation day of the stress treatment (10^7^–10^8^ CFU/g of root dry weight).

### Moderate Water-Deficit Experiment

**FIGURE 2:**
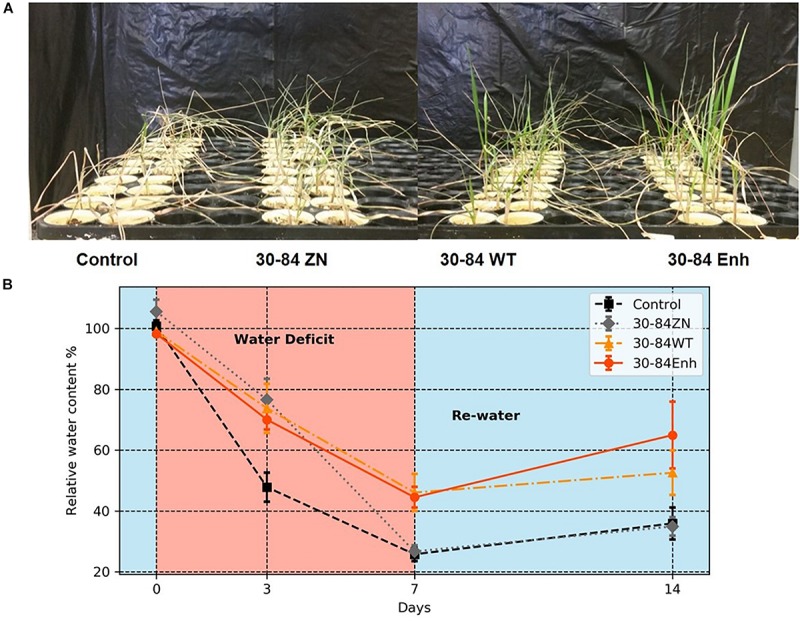
Colonization by phenazine-producing strains reduced plant drought stress symptoms and enhanced whole plant relative water content. Wheat seedlings were grown unstressed for 21 days in cones containing autoclaved soil inoculated either with 30-84Enh, 30-84WT, or 30-84ZN, or no inoculum (control). Following this establishment period, plants were not watered for 7 days. All plants were then re-watered for 7 days at 3-day intervals. **(A)** Pictures of seedlings on Day 7 of the water deficit in the moderate water-deficit experiment. **(B)** Relative water content of above ground plant tissue was measured: before water deficit (Day 0), during water deficit (Days 3 and 7), and 7 days after re-watering (Day 14). Data are the mean and standard errors (*N* = 6 plants).

Seedlings colonized by the different bacterial treatments experienced the water deficit differently as is apparent from the degree of plant wilting and leaf browning observed following 7 days without water (Figure 2A). Relative water content (RWC) of seedlings provided a quantifiable estimate of these differences (Figure 2B). After just 3 days, the RWC was found to be significantly lower in the no-inoculum control seedlings (48 ± 5%) compared to the bacterially colonized seedlings (73 ± 8, 70 ± 3, 77 ± 7%, for 30-84WT, 30-84Enh, and 30-84ZN, respectively). By day 7, there were significant differences in RWC between the seedlings colonized by phenazine-producing strains (46 ± 6, 45 ± 3%, for 30-84WT and 30-84Enh, respectively) compared to 30-84ZN (27 ± 2%) or the no-inoculum controls (26 ± 2%).

**FIGURE 3:**
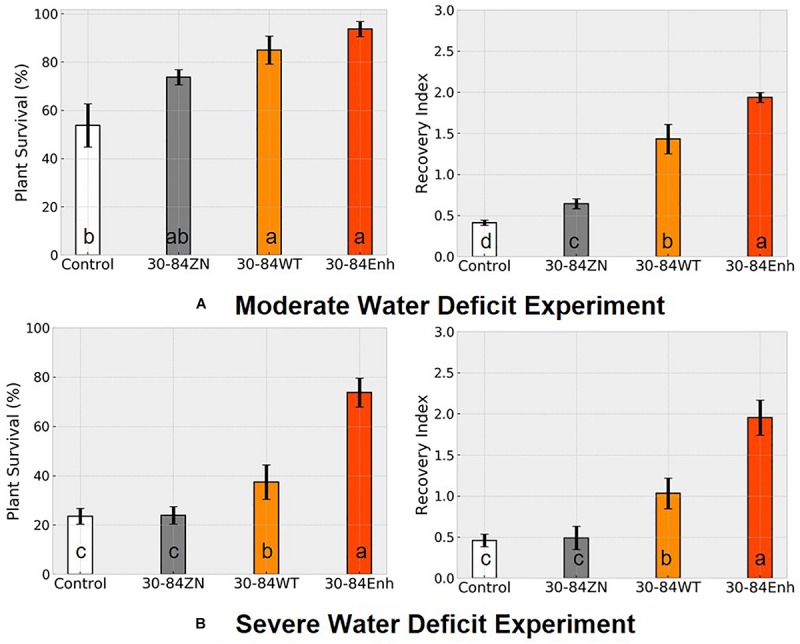
Colonization by phenazine-producing strains improved seedling survival and recovery from drought stress. Wheat seedlings were grown unstressed for 21 days in cones containing autoclaved soil inoculated either with 30-84Enh, 30-84WT, or 30-84ZN, or no inoculum (control). After this establishment period, plants were not watered for 7 days (**A**, moderate water-deficit experiment) or 10 days (**B**, severe water-deficit experiment). All plants were then watered and survival and recovery index were determined after 7 days of well-watered conditions. Survival was expressed as the percentage of plants surviving the Moderate or Severe water-deficit after 7 days of recovery. Recovery Index was based on the amount of above ground tissue recuperated (RI-0 = no recovery, RI-1 = slight new growth, RI-2 = recovery of partial leaf, RI-3 = recovery of one or more entire leaves). Data are the means and standard errors (Blocks = 4, Reps = 20). Values with the same letter do not differ statistically as determined by a Fishers protected Least Significantly Difference (LSD) test (*P* > 0.05).

Following the water deficit interval, all seedlings were watered and after 7 days, plant recovery index (RI) and survival rate were determined for each treatment. RI was based on the amount of above ground tissue recuperated after the 7-day recovery. After 7 days of recovery, survival of seedlings colonized by phenazine-producing strains exceeded 80% (85 ± 6, 94 ± 3%, for 30-84WT and 30-84Enh, respectively), whereas survival of the no-inoculum controls was significantly less (54 ± 6%); survival of 30-84ZN colonized seedlings was intermediate (74 ± 3%) (Figure 3A). Seedlings colonized by both phenazine-producers recovered better than 30-84ZN or the no-inoculum control plants. RI values followed this trend: 30-84Enh > 30-84WT > 30-84ZN > no-inoculum control (Figure 3A). Mean RWC of surviving seedlings after the 7 days recovery period showed the similar trend (65 ± 11, 54 ± 7, 35 ± 3, and 36 ± 5%, respectively (Figure 2B).

Following the seven day recovery period, the seedlings were exposed to a second cycle of water deficit (7 days) and recovery (7 days). After the second cycle, overall survival (across all blocks) of the starting number of seedlings colonized by 30-84Enh and 30-84WT were 49 and 45%, respectively, compared to 8% for the no-inoculum control seedlings; survival of seedlings colonized by 30-84ZN was 38% (data not shown). These results suggest that the effects of bacterial phenazine production on drought-stress tolerance and recovery contributed to seedling resilience to repeated water deficit cycles.

### Severe Water-Deficit Experiment

Although seedling survival was greatest for plants colonized by phenazine producers, seedling survival for all treatments was lower than in the moderate water-deficit experiment after one drought/recovery cycle. Survival rates for the no-inoculum controls and seedlings colonized by phenazine deficient (ZN) colonized seedlings were 24 ± 4 and 25 ± 3%, respectively, compared to 42 ± 7% by the 30-84WT and 74 ± 5% for 30-84Enh colonized (Figure 3B). As in the moderate water-deficit experiment, seedlings colonized by either of the phenazine-producers recovered better than seedlings colonized by 30-84ZN or the no-inoculum control plants. RI values were significantly higher for the seedlings colonized by the phenazine-producing strains compared to either of the controls (Figure 3B). Following the 7-day recovery period, the plants inoculated with wild type had an average recovery index of 1, indicating that most seedlings had modest regrowth. Seedlings inoculated with the enhanced phenazine-producer had an average recovery index of 2 indicating that on average, seedlings had partial to complete recovery of leaves. Seedlings colonized by the phenazine deficient mutant (30-84ZN) or no-inoculum controls had an average recovery index of 0.5, indicating that most seedlings did not recover at all or had only slight regrowth, usually restricted to near the crown. Mean bacterial population densities were measured on day four of the recovery period and were 10^5^–10^6^ CFU/g of root dry weight with no significant differences among treatments. Given the severity of the first drought stress, it was difficult to unambiguously determine survival or recovery after the second drought/recovery cycle (data not shown).

### Effects on RSA in Non-vernalized vs. Vernalized Plants

Because TAM 112 is a winter wheat variety, vernalization is needed to promote plant development beyond the vegetative stage. The effects of bacterial colonization on the growth and development of non-vernalized seedlings and vernalized plants grown to jointing stage in response to severe water-deficit were compared.

**FIGURE 4:**
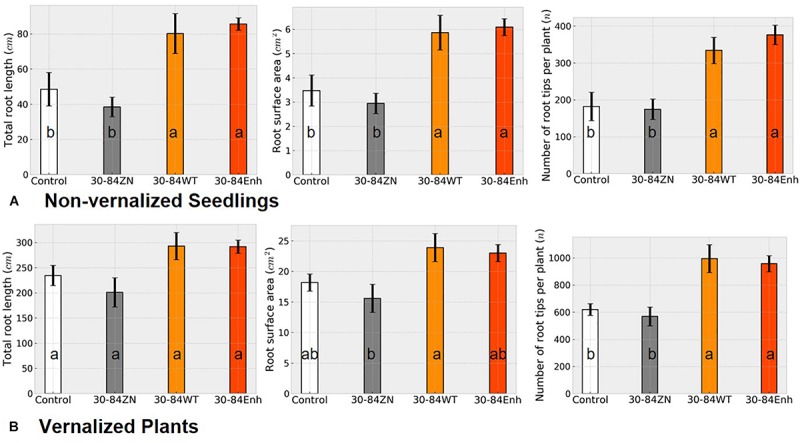
Severe water-deficit experiment: effect of phenazine-producing strains on root system development. **(A)** Non-vernalized seedlings and **(B)** vernalized plants were grown in autoclaved soil inoculated either with 30-84Enh, 30-84WT, or 30-84ZN, or no inoculum (control). Non-vernalized seedlings were grown 21 days under well-watered conditions and then exposed to two water-stressed cycles (10 and 7 days); Vernalized seedlings were grown to the jointing stage under well-watered conditions and then exposed to a 15-day water deficit. Root system metrics were obtained from WinRhizo software including total root length, root surface area, and number of root tips per plant. These experiments were repeated once. Data are the mean and standard errors. Values with the same letter do not differ statistically as determined by a Fishers protected Tukey test (*P* > 0.05), *N* = 5 plants (two replicate plants per scan).

Changes in RSA as a result of bacterial treatment for both non-vernalized seedlings and vernalized plants grown under water-limited conditions in plastic tubes were similar to each other (Figure 4) as well as to the changes in RSA observed in the well-watered, seedlings grown in pots (Figure 1). At the end of the severe water deficit, the roots of non-vernalized plants colonized by the phenazine-producing strains had significantly greater total root length and surface area and more root tips (>1.5 fold more) than plants colonized by 30-84ZN or the no-inoculum controls (Figure 4A). Vernalized plants colonized by the phenazine-producing strains also had significantly more root tips (>1.5 fold more) than plants colonized by 30-84ZN or no-inoculum controls (Figure 4B). Additionally, there was a trend toward greater root length and surface area for the vernalized plants colonized by the phenazine-producing strains. However, given the larger size of the root system and the higher variability among plants related to the effects of severe water deficit these differences were not statistically significant. These results suggest that as the size of the root system increases with plant age, any bacterially mediated effects on total root length and surface area become less pronounced, whereas the effect on the proliferation of higher order roots and root tips remains clearly discernable.

### Production of Different Phenazines by *P. chlororaphis* 30-84 Also Promotes Root Growth of Wheat Seedlings in Natural Soil

To determine whether different types of phenazines promote root growth in natural soil, root production was measured during repeated plant/harvest cycles as described previously (Yu et al., 2018a). The bacterial strains used in this experiment included 30-84Enh, 30-84WT, and 30-84ZN, as well as 30-84MS (produces pyocyanin) and 30-84H (produces phenazine-1-carboxamide). The phenazine non-producer 30-84ZN was included as the negative control. Repeated harvest cycles were used to select for naturally occurring strains that are good wheat rhizosphere colonists thereby increasing rhizosphere competition. Root colonization by all phenazine-producing strains was consistent at ∼10^7^ CFU/g root dry weight at every harvest cycle. The results indicated that the dry weight root production of seedlings colonized by all phenazine-producing derivatives of *P. chlororaphis* 30-84 was significantly greater than that of seedlings colonized by 30-84ZN at most harvest cycles (Table 2). In this experiment, by having isogenic derivatives of *P. chlororaphis* 30-84 that produced different phenazines we tested whether changes in the root system of seedlings were related specifically to differences in the structure of the phenazine produced by the bacterial colonist (rather than differences between species that produce different phenazines). These results demonstrated that different phenazines may stimulate seedling root growth, and that these results may occur even in competition with the natural soil microbiome.

**TABLE 2:** Production of different phenazines by *P. chlororaphis* 30-84 promotes root growth of wheat seedlings in natural soil.

**Strain Derivative**	**Cycle 1 Root dry weight (mg)**	**Cycle 2 Root dry weight (mg)**	**Cycle 3 Root dry weight (mg)**	**Cycle 4 Root dry weight (mg)**	**Cycle 5 Root dry weight (mg)**
30-84Enh	1.15 ± 0.19^a^	0.96 ± 0.13^a^	0.92 ± 0.15^a^	0.92 ± 0.11^a^	1.00 ± 0.13^a^
30-84WT	1.22 ± 0.11^a^	1.13 ± 0.14^a^	0.97 ± 0.08^a^	0.94 ± 0.04^a^	0.87 ± 0.08^a^
30-84MS	1.14 ± 0.14^a^	1.14 ± 0.14^a^	1.18 ± 0.16^a^	0.97 ± 0.07^a^	0.98 ± 0.07^a^
30-84H	0.89 ± 0.14^ab^	0.79 ± 0.14^ab^	0.71 ± 0.09^ab^	0.89 ± 0.14^a^	0.85 ± 0.06^a^
30-84ZN	0.82 ± 0.07^b^	0.69 ± 0.03^b^	0.68 ± 0.04^b^	0.60 ± 0.07^b^	0.59 ± 0.03^b^

*Wheat seedlings were pre-germinated 3 days and then grown in cones containing natural (not autoclaved) soil inoculated either with 30-84Enh, 30-84WT, 30-84MS (produces pyocyanin), 30-84H (produces phenazine-1-carboxamide), or 30-84ZN (no phenazine control). Seedlings were grown under well-watered conditions for 21 days, and 6 plants per treatment were harvested at the end of each plant/harvest cycle. Bacterial populations on roots were determined by serial dilution plating on LB amended with rifampicin and cycloheximide, and root dry weight biomass was determined. For the plants not used in the CFU estimations, shoots were removed at the soil surface level. The soil and remaining root systems were homogenized and reused as planting medium, as previously described (Yu et al., 2018a). Newly germinated wheat seedlings were re-planted into the processed soil and the plant/harvest/sample cycle was repeated 5 times. Data are the mean dry weight biomass and standard error (*N* = 6). For each plant/harvest cycle, treatments with the same letter are not significantly different.*

## Discussion

This study demonstrated that phenazine-producing *Pseudo- monas* promote drought-stress tolerance and resilience to repeated cycles of water deficit in seedlings of the winter wheat cultivar TAM112, a cultivar specifically selected for drought-stress tolerance. The focus was on improved drought-stress tolerance in seedlings because seedling establishment may have large impacts on crop stand and yield. Investigation of the specific role of bacterial phenazine production in seedling drought-stress tolerance was facilitated by having isogenic derivative strains of *P. chlororaphis* 30-84 that were unable to produce phenazines or produced more phenazine than the wild type. For the assays, bacterial inoculum was applied to soil and bacteria colonized the developing seedling root systems, reaching similar densities. Seedlings colonized by phenazine-producing strains (wild type and 30-84Enh) had significantly greater survival and recovery from both the first and second water deficits compared to seedlings colonized by the phenazine non-producing strain or the no-inoculum controls, indicating phenazine production promotes drought stress resilience to repeated cycles of water deficit. Moreover, the differences in plant phenotypes were more pronounced when comparing seedlings treated with the enhanced phenazine producer and no-inoculum controls (Figure 3).

It was evident that seedlings colonized by phenazine-producing strains experienced the severity of the water-deficit differently from seedlings that were not colonized by them. After withholding water for 7 days, most of the no-inoculum control seedlings wilted to collapse (the basis for the selection of 7-day moderate water-deficit treatment) whereas the phenazine-treated seedlings displayed less severe drought-stress symptoms (Figure 2). As the water deficit progressed, the relative water content of seedlings colonized by *P. chlororaphis* 30-84 and specifically the phenazine-producing strains remained higher than for seedlings that were not treated with bacteria. After 7 days recovery, although there was significant variability among seedlings in RWC, average RWC of surviving seedlings colonized by phenazine-producers was >50% as compared to ∼35% for seedlings colonized by 30-84ZN or the no-inoculum controls. These data indicate that seedlings colonized by the phenazine-producing strains suffered less from dehydration during the water deficit and recovered better, potentially contributing to better resilience during the second drought/recovery cycle. For the severe water-deficit experiment, water was withheld until all treatments experienced severe drought-stress symptoms (collapsed). Following the 7-day recovery period, the plants inoculated with wild type had modest regrowth, whereas seedlings inoculated with the enhanced phenazine-producer had partial to complete recovery of leaves. In contrast, seedlings colonized by the phenazine deficient mutant (30-84ZN) or no-inoculum controls did not recover at all or had only slight regrowth, usually restricted to the crown. These results indicate that the delay in the onset of drought symptoms experienced by seedlings colonized by the phenazine-producing strains enabled the seedlings to survive and recover better from the 10-day water deficit, which was sufficiently severe that all seedlings displayed severe wilt symptoms.

Measurements related to seedling RSA were compared among treatments in order to investigate what root system developmental phenotypes may have contributed to the enhanced drought-stress avoidance phenotype of seedlings colonized by the phenazine-producing strains (Figure 1). Because more than 95% of seedling water uptake occurs at the root tips via root hairs, greater investment in linear root length and surface area (related to greater soil exploration) and proliferation of higher order roots (resulting in more root tips) are traits that are commonly associated with drought-stress avoidance phenotypes (Comas et al., 2013). Greater surface area and more tips also provide greater root-rhizosphere interface for recruitment of and interactions with soilborne microorganisms. Under well-watered conditions, seedlings with or without bacterial treatment generally attained the same total dry weight, indicating no initial overall growth promoting effect due to colonization by the bacteria. However, seedlings colonized by phenazine-producing strains invested more (in terms of dry weight) in the root system than the shoot system, and this increased investment in roots was most pronounced for seedlings colonized by the phenazine overproducer 30-84Enh. Greater investment in roots by seedlings colonized by the phenazine-producing strains generally translated into greater linear root length and surface area and number of root tips, compared to seedlings colonized by 30-84ZN or the no-inoculum controls. The most prominent difference among seedlings was in the number of root tips, with seedlings colonized by the wild type or the phenazine-overproducer 30-84Enh having ∼1.5 to 2-fold more root tips, respectively, than the no-inoculum controls. It is now well recognized that short-term water deficit often results in increased root production at the expense of shoot production, which may reflect an attempt to shift growth allocation toward acquisition of resources that are most limiting (Comas et al., 2013; Xu et al., 2015). Production of new root tips via proliferation of higher order roots may be more important for the uptake of water and nutrients than the total amount of root length and surface area (Robinson et al., 1991; Comas et al., 2013). Given the importance of root tips for water uptake capacity, it is likely that this developmental phenotype in seedlings colonized by phenazine-producing strains primes root systems for better tolerance of water deficit conditions.

We also compared RSA for seedlings grown with or without phenazine-producers under water deficit conditions to see whether drought stress would equalize or amplify differences in the root system phenotypes we observed for seedlings grown under well-watered conditions (Figure 4). Similar to the seedlings grown without water deficit, after two cycles of 10-day water deficit and recovery, seedlings colonized by phenazine-producing strains 30-84Enh and had almost 1.5 to 2-fold more root tips than seedlings colonized by 30-84ZN or the no-inoculum controls. For vernalized plants grown with a 15-day water deficit, colonization by phenazine producers resulted in an average of ∼1000 root tips compared to an average ∼600 root tips within root systems of seedlings colonized by 30-84ZN or the no-inoculum controls. The root systems of non-vernalized seedlings colonized by the phenazine-producing strains also had greater linear length and surface area than the no-inoculum controls. A similar trend was observed for the root systems of the larger vernalized plants, although this was not significant. As seedlings develop and root systems become larger, new growth becomes an increasingly smaller percentage of the established root system. This may explain why apparent differences in root system development among older seedlings having different inoculation treatments were not significantly different. Although none of the seedlings appeared container bound, restrictions on root growth imposed by container size and shape may affect RSA and thus limit root development. Nonetheless, the results clearly demonstrate the effect of the phenazine-producing strains in stimulating root system growth and potentially water uptake capacity in seedlings and larger vernalized plants.

Previous observations reported that indigenous pseudomonads having phenazine biosynthetic genes were detected at higher frequencies and in greater numbers on roots of winter wheat grown in dryland production as compared to irrigated production on the Columbia Plateau in Eastern Washington (Mavrodi D.V. et al., 2012; Mavrodi O.V. et al., 2012). Moreover, PCA was detected in the rhizosphere of field-grown wheat at nanomolar concentrations and in direct proportion to the population density of pseudomonads having phenazine biosynthetic genes (Mavrodi D.V. et al., 2012). More recently, Mavrodi et al. (2018) demonstrated that the frequency and size of rhizosphere populations of indigenous pseudomonads having phenazine biosynthetic genes was reduced on plots within a dryland production site receiving 3 years of overhead irrigation as compared to adjacent non-irrigated plots. Subsequent work by Le Tourneau et al. (2018) used mesocosms inoculated with the phenazine-producing strain *Pseudomonas synxantha* 2–79RN_10_ maintained at moisture levels approximating those found in dryland or irrigated wheat fields to investigate phenazine production. They showed that although population−level expression of PCA biosynthesis genes was decreased under dryland conditions, the expression of these genes was upregulated relative to background, indicating the importance of phenazine biosynthesis to cells under dryland conditions. Studies in Texas setting the stage for the present research (Mahmoudi, 2017) indicated that populations of native pseudomonads having phenazine biosynthetic genes were generally higher in the rhizospheres of the drought-tolerant cultivars TAM 111 and TAM 112 compared to drought-sensitive TAM 304. This was especially true when the cultivars were grown in soil collected from a long-term dryland production site, compared to soil collected from an adjacent long-term irrigation production site. On TAM 111 and 112 roots, native bacteria having phenazine biosynthetic genes typically reached populations sizes above 10^5^ CFU/g of root (Mahmoudi, 2017), a threshold density considered necessary for biologically activity in the rhizosphere (Pierson et al., 1994; Khan et al., 2005; Maddula et al., 2006). In contrast, only some roots of the drought sensitive cultivar TAM 304 had detectable levels of bacteria having phenazine biosynthetic genes. In both the Washington and Texas studies, results were based on frequency of detection of a phenazine biosynthetic gene and thus provide no information on the specific types of phenazines that may be produced. So which phenazines are important for mediating the enhanced-root growth phenotype? Only those specific phenazines produced by *P. chlororaphis* 30-84 (e.g., PCA, 2OHPCA or 2OHPHZ), or do other phenazines have similar root-growth promoting effects? In the present study, we addressed the question using isogenic strains of *P. chlororaphis* 30-84 capable of producing structurally different phenazines. Our results indicated that relative to the phenazine non-producing strain, production of pyocyanin and to a lesser extent phenazine-1-carboxamide also promoted seedling root-growth (Table 2). These findings suggest that growth promotion is not limited to a specific type of phenazine. The present study, albeit limited to container experiments, suggests that in addition to seedling disease suppression, phenazine producers play additional ecological roles such as priming seedling root system development for improved drought-stress tolerance.

It is unclear whether phenazine production by rhizosphere colonizing bacteria contributes to plant growth and development *directly* via plant growth promoting activities, *indirectly* via modifications to the rhizosphere environment, or *both*. For example, in addition to suppression of seedling disease, plant growth-promoting bacteria have been reported to contribute to drought tolerance directly via a number of mechanisms including promoting changes in plant growth and development, altering stomatal regulation, producing compatible solutes, mediating redox stress, producing or catabolizing metabolites or volatiles that act as plant hormones, or tapping into other plant stress response pathways (Berendsen et al., 2012; Farag et al., 2013; Nadeem et al., 2014; Ngumbi and Kloepper, 2016; Naylor and Coleman-Derr, 2018). *P. chlororaphis* 30-84 (including 30-84ZN) produces indole acetic acid and ACC-deaminase *in vitro* (data not shown). Production of these metabolites in the rhizosphere potentially could contribute to lateral root growth or reduce the ethylene levels (which may increase in response to drought-stress) that can be an inhibitor of plant growth, respectively (Glick, 2014). Interestingly, *P. chlororaphis* O6, which produces the same phenazines as *P. chlororaphis* 30-84, also produces the volatile organic compound (VOC) 2R,3R-butanediol. Production of this VOC by *P. chlororaphis* O6 was found to be a major determinant in inducing plant drought-stress tolerance in *Arabidopsis thaliana*, though the contribution of phenazine production to drought-stress tolerance was not tested (Cho et al., 2008). Although all of these mechanisms may be important contributors to drought-stress tolerance, they would not explain the benefits conferred to plants by phenazine-producing bacteria.

Phenazines as redox active molecules may contribute to ROS-responsive signaling pathways responsible for stress tolerance in plants. Increasingly, management of redox homeostasis has been implicated as a significant contributor to plant stress management, and the expression of genes encoding ROS-scavenging enzymes, antioxidant biosynthesis, and ROS system regulation have been shown to play important roles in plant response to water deficit by stimulating/modulating global plant stress responses (Das P. et al., 2015; Sewelam et al., 2016). It has been suggested that alterations in systemic ROS signals such as generated after pathogen encounter (or in this case generation of ROS at the root surface via the production of phenazines) may alter water relations and salt uptake through their effects on water and ion transport (Kissoudis et al., 2014). Additionally, lateral root initiation and emergence is regulated by auxin and ROS signaling i.e., peroxidase activity in response to hydrogen peroxide accumulation in lateral root primordia may facilitate the transition to cell differentiation (Casimiro et al., 2001; Manzano et al., 2014). Thus, the production of redox active phenazines may serve to both increase the availability of ROS and induce plant ROS system regulation, leading to drought stress response priming and/or increased lateral root production.

Phenazine production by rhizosphere colonizing bacteria may contribute to plant growth and development *indirectly*. Bacterial extracellular polysaccharide production has been shown to provide significant indirect benefits to plant growth and development via improved soil structure and increased soil water retention (Chang et al., 2007; Naseem and Bano, 2014). These improvements coupled with high microbial respiration rates and associated water release by rhizosphere microbial communities may lead to further improvements in water availability, therein enabling plants to avoid drought stress longer. Previous research demonstrated phenazine production was required for measurable extracellular matrix production, i.e., extracellular matrix production by 30-84ZN was almost nonexistent (Wang et al., 2016). More recently, Le Tourneau et al. (2018) demonstrated that as compared with an isogenic PCA deficient mutant strain, PCA-producing *P. synxantha* 2-79RN_10_ promoted biofilm development in dryland wheat rhizospheres. It is likely that extracellular matrix production by phenazine-producing bacteria functions as a humectant that increases water availability in the rhizosphere, thus enabling a longer adjustment period for plant water-deficit stress responses and a more pronounced drought stress avoidance phenotype.

These results using phenazine deficient and overexpressing derivatives explicitly demonstrate that phenazine-producing rhizobacteria significantly increase the tolerance and resilience of wheat seedlings to severe water-deficit, at least in part by influencing increased root system investment and branching, resulting in a doubling of the number of available root tips for water and nutrient uptake. However, as indicated from previous work showing the broad transcriptomic consequences associated with the lack or overexpression of phenazines (Wang et al., 2016), we recognize that the microbial mechanisms underlying these results may be more complex i.e., not solely due to phenazine-specific effects. Nevertheless, the prevalence of bacteria carrying the genes for phenazine production in dryland wheat soils and frequency of occurrence on the roots of wheat cultivars bred for dryland agriculture suggest that phenazine-producers may be recruited by wheat under dryland conditions. Future work should focus on breeding wheat lines capable of taking full advantage of this microbial functionality in dryland soils to improve drought-stress tolerance.

## Data Availability

The datasets generated for this study are available on request to the corresponding author.

## Author Contributions

TM and JY carried out the experiments and performed the data analysis. TM, JY, LP, and EP contributed to the interpretation of results and writing and revisions of the manuscript. EP provided project supervision and major contribution to the final draft. All authors contributed to the conceptualization and experimental design of the study, and read and approved the submitted version.

## Conflict of Interest Statement

The authors declare that the research was conducted in the absence of any commercial or financial relationships that could be construed as a potential conflict of interest.

## Footnotes

1 https://soilseries.sc.egov.usda.gov/OSD_Docs/P/PULLMAN.html
